# Supplementary material for: High Serum Phosphate Is Associated with Cardiovascular Mortality and Subclinical Coronary Atherosclerosis: Systematic Review and Meta-Analysis
Source: Nutrients. 2024 May 24;16(11):1599. doi: 10.3390/nu16111599 (PMC11174514; doi:10.3390/nu16111599)
Supplement: Supplementary file 1 [file nutrients-16-01599-s001.zip › nutrients-2978852-supplementary.pdf]

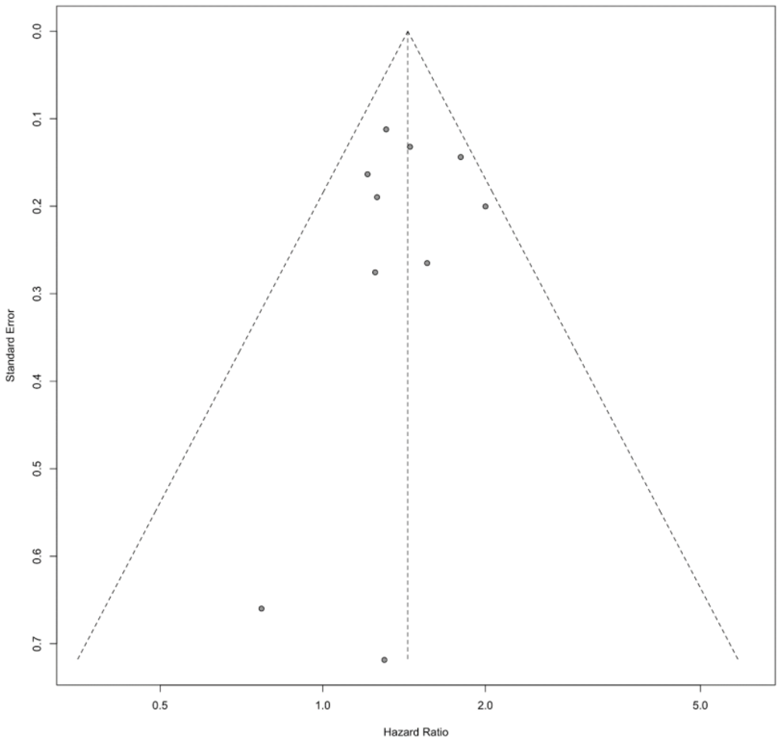

Supplementary Figure S1. Funnel plot of association between serum phosphate and cardiovascular mortality.

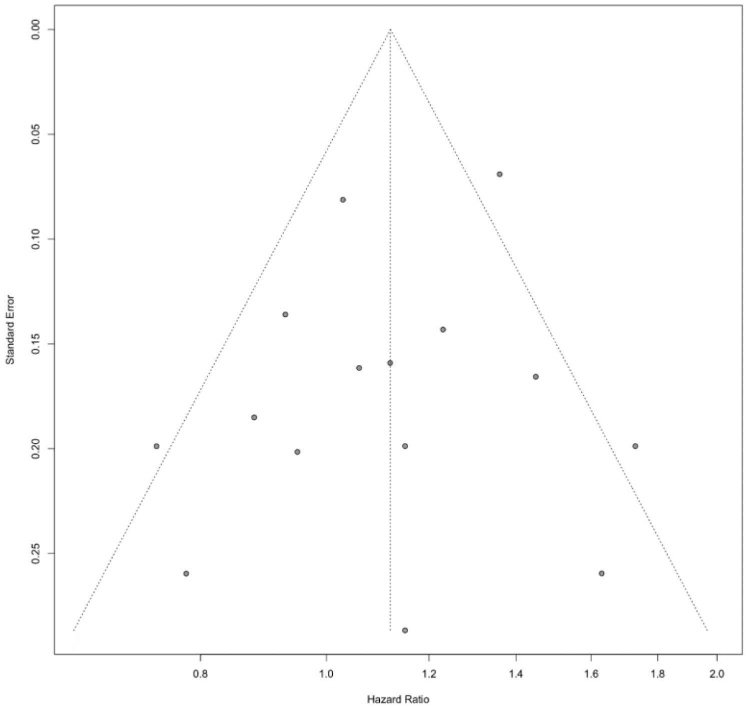

Supplementary Figure S2. Funnel plot of the association between serum phosphate and cardiovascular disease.

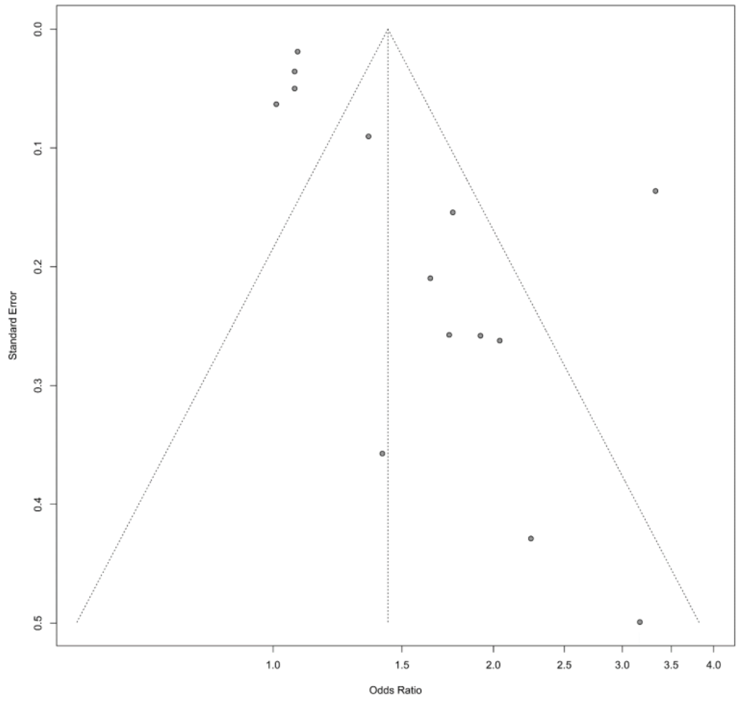

Supplementary Figure S3. Funnel plot of association between serum phosphate and subclinical coronary atherosclerosis.
